# The impact of fluticasone furoate/vilanterol on healthcare resource utilisation in the Salford Lung Study in chronic obstructive pulmonary disease

**DOI:** 10.1177/17534666211001013

**Published:** 2021-03-29

**Authors:** Nawar Diar Bakerly, Dominy Browning, Isabelle Boucot, Jodie Crawford, Sheila McCorkindale, Norman Stein, John P. New

**Affiliations:** Department of Respiratory Medicine, Salford Royal NHS Foundation Trust, Stott Lane, Salford M6 8HD, UK; Manchester Academic Health Sciences Centre, University of Manchester, Citylabs 1.0, Nelson Street, Manchester M13 9NQ, UK; Respiratory Research and Development, GlaxoSmithKline plc., Brentford, Middlesex, UK; Global Respiratory Therapy Area, GlaxoSmithKline plc., Brentford, Middlesex, UK; Clinical Statistics, GlaxoSmithKline plc., Stockley Park West, Uxbridge, Middlesex, UK; NIHR Clinical Research Network Greater Manchester, Citylabs 1.0, Manchester, UK; Manchester Academic Health Sciences Centre, University of Manchester, Citylabs 1.0, Manchester; NorthWest EHealth, Manchester Science Park, Manchester, UK; Department of Respiratory Medicine, Salford Royal NHS Foundation Trust, Salford, UK; Manchester Academic Health Sciences Centre, University of Manchester, Citylabs 1.0, Manchester, UK

**Keywords:** COPD, effectiveness, fluticasone furoate, healthcare resource utilisation, usual care, vilanterol

## Abstract

**Aim:**

The Salford Lung Study (SLS) in chronic obstructive pulmonary disease (COPD) was a randomised controlled trial evaluating the effectiveness and safety of initiating fluticasone furoate/vilanterol (FF/VI) 100/25 µg *versus* continuing usual care (UC) in patients with COPD and a history of exacerbations. Here, we investigate the impact of initiating FF/VI on healthcare resource utilisation (HRU) in SLS COPD.

**Methods:**

HRU and interventions were determined from patients’ electronic health records. Annual rates of on-treatment all-cause and COPD-related secondary care contacts (SCCs) and primary care contacts (PCCs) for FF/VI *versus* UC were analysed using a general linear model. Costs were derived from national data sources.

**Results:**

Least-squares (LS) mean annual rates of all-cause (9.81 *versus* 9.36) and COPD-related (1.57 *versus* 1.48) SCCs were similar for FF/VI and UC, as were rates of all-cause hospitalisations (0.87 *versus* 0.82). Mean duration of hospital stay/patient was 4.5 and 4.2 days, respectively. COPD-related SCC mean total cost/patient was £484 FF/VI and £475 UC. LS mean annual rates of all-cause PCCs were significantly higher for FF/VI (21.20 *versus* 18.88 UC; *p* < 0.001). LS mean annual rates of COPD-related PCCs were similar for FF/VI and UC (2.42 *versus* 2.46). All-cause PCC mean total cost/patient was £900 FF/VI *versus* £811 UC, but COPD-related PCC costs were similar (£116 *versus* £114). Direct COPD-related total medical costs/patient were significantly lower for FF/VI (LS geometric mean £806 *versus* £963 UC; *p* < 0.001).

**Discussion:**

In patients with COPD and exacerbation history, FF/VI may represent a less costly alternative to current therapies.

GlaxoSmithKline plc. study HZC115151; ClinicalTrials.gov NCT01551758.

*The reviews of this paper are available via the supplemental material section.*

## Introduction

Chronic obstructive pulmonary disease (COPD) is the fourth leading cause of mortality worldwide, accounting for ~6% of all deaths globally in 2012.^1^ COPD is a major cause of chronic morbidity, and the prevalence and burden of this disease is projected to increase due to continued exposure to COPD risk factors and an increasing elderly population.^1^ The economic burden of COPD is substantial, with over half of the European Union’s respiratory disease costs consumed directly by this disease (38.6 billion Euros).^1^ There are also considerable indirect social costs of COPD, including those incurred by disability and work absence. Thus, there is a need to improve treatment options for patients with COPD and reduce the economic burden associated with this disease.

The efficacy and safety of new medicines is routinely assessed in double-blind randomised controlled trials (DBRCTs). Due to stringent inclusion/exclusion criteria, such trials typically enroll highly selected patient cohorts that are not necessarily representative of the wider disease population in routine care.^2–7^ Furthermore, patients in DBRCTs are typically followed up according to rigorous protocols that are not reflective of everyday clinical care. This underscores the need for evaluation of new treatments in well-designed effectiveness trials, involving relevant patient populations in settings that represent everyday clinical practice.^7–9^

The Salford Lung Study in COPD (SLS COPD; ClinicalTrials.gov identifier: NCT01551758; GlaxoSmithKline plc. study HZC115151) was a prospective, 12-month, open-label, randomised controlled trial conducted in UK primary care, evaluating the effectiveness and safety of initiating once-daily inhaled fluticasone furoate/vilanterol (FF/VI) 100/25 µg *versus* continuing usual care (UC) in patients with COPD and a history of exacerbations. The trial was designed to provide additional information on the benefit–risk profile of FF/VI in a broad population of patients with COPD who were representative of those in everyday clinical practice. The primary endpoint was the mean annual rate of moderate/severe exacerbations among patients experiencing an exacerbation within 1 year before the trial. In the primary analysis of SLS COPD, once-daily treatment with FF/VI was associated with a lower rate of exacerbations than UC, without a greater risk of serious adverse events.^10^

Here, we aim to provide a detailed description of the impact on healthcare resource utilisation (HRU) of initiating FF/VI 100/25 µg *versus* continuing UC in everyday clinical practice in SLS COPD, and to provide detailed analyses of real-world HRU and the costs of managing COPD within the UK National Health Service (NHS).

## Methods

### Study design and patients

The SLS COPD study design has been reported previously,^10,11^ and is briefly described in the online Supplemental Material.

The SLS COPD study was designed to minimise interference with patients’ and physicians’ behaviours regarding COPD management. Data were captured *via* patients’ electronic health records (EHRs) using an integrated primary and secondary care-linked database system developed by NorthWest EHealth that allowed for remote, real-time collection and monitoring of study data without disrupting patients’ normal contact with their healthcare professionals (HCPs).^10–12^ This also provided a real-world picture of HRU in a heterogeneous and representative COPD patient population.

All patients in SLS COPD provided written informed consent for participation. The trial protocols were approved by the National Research Ethics Service Committee North West, Greater Manchester South (approval numbers 11/NW/0798 and 12/NW/0455).

### Study outcomes

#### HRU endpoints

SLS COPD incorporated several prespecified HRU endpoints, including all-cause and COPD-related secondary care contacts (SCCs) and primary care contacts (PCCs) (secondary endpoints). Number of hospitalisations and total number of days in hospital were also included.^10,11^

#### Definition of on-treatment SCCs and PCCs

On-treatment SCCs and PCCs were healthcare contacts documented during the treatment period [i.e. from the start date to the stop date of treatment exposure + 1 day (date of study discontinuation)].

SCCs were defined as any specialist (outpatient) care, accident and emergency (A&E) visit, or inpatient admission, on a given date. Patients who had an A&E contact and subsequent inpatient admission were recorded as having two separate SCCs. Another assumption was that an episode in critical care could be linked to an inpatient admission event. COPD-related SCCs were defined using codes or speciality descriptions recorded in patients’ EHRs. Respiratory-related SCCs were based on a prespecified list of speciality descriptions and national diagnosis codes; these were included in the COPD-related definition. Hospital admissions were considered to be COPD related based on a prespecified list of International Statistical Classification of Diseases and Related Health Problems 10th Revision (2010) (ICD-10) codes.^13^

PCCs were defined as any encounter between a patient and a general practitioner, nurse, or other HCP working in the NHS on a given date (including telephone contacts; not including protocol-defined, study-related visits). PCCs were classified as COPD related if the most prominent signs and symptoms were a direct result of COPD, according to a prespecified list of Read codes. COPD-related PCCs that occurred on a date when the patient was seen by more than one type of HCP were assigned to all of the roles on that date due to a lack of information required to link specific Read codes to HCP roles.

#### HRU data collection and extraction

Data were collected electronically *via* extracts from the integrated primary and secondary care-linked database system onto the central NorthWest EHealth study server for analysis.

#### Unit costs for HRU and medications

Prices were derived for general practice resource use from the Personal Social Services Research Unit,^14^ Monthly Index of Medical Specialities for prescription use,^15^ and NHS Reference Costs for hospital attendance.^16,17^ Supplemental Table 1 summarises unit costs for HRU and study medications. Exacerbation costs were defined as any costs incurred between an exacerbation start date and end date. Cost calculations are described in the online Supplemental Material.

### Statistical analyses

HRU and cost analyses were conducted in the intent-to-treat (ITT) population, which comprised all randomised patients who received at least one prescription of study medication. All costs (except unit costs) were to the nearest whole pound [GBP (£), 2014–2015]. Supporting analyses were conducted in the primary effectiveness analysis (PEA) population, which comprised a subset of ITT patients who had experienced at least one moderate/severe COPD exacerbation in the prior year. Results are reported for a time horizon of 1 year and from an NHS perspective.

All analyses were prespecified, except for *post hoc* analyses of: overall mean COPD Assessment Test™ (CAT) score at baseline; all-cause costs in secondary care; cost of COPD exacerbations; COPD-related hospital admissions; and rates of on-treatment PCCs, which were conducted using a revised categorisation of HCP-seeing patient and excluding study-related Read codes. The rationale for this was that previously published data on annual rates of PCCs in SLS COPD^10^ included miscoded administrative procedures and study-related visits. Adjusted all-cause PCC values were calculated *post hoc* to more accurately reflect PCC rates.

Annual rates of on-treatment all-cause and COPD-related SCCs and PCCs were analysed using a general linear model, assuming an underlying negative binomial distribution adjusting for randomised treatment, baseline COPD maintenance therapy per randomisation stratification, number of moderate-to-severe COPD exacerbations in the year prior to randomisation (<2 or ⩾2), smoking status at baseline, and logarithm of time on treatment as an offset variable.

## Results

### Patient demographics and baseline characteristics

Overall, 2802 patients were randomised in SLS COPD, and 2799 were included in the ITT population (FF/VI, *n* = 1396; UC, *n* = 1403). Patient demographics and baseline characteristics have been reported previously^10^ and are summarised in Table 1.

**Table 1.** Patient demographics and baseline clinical characteristics (ITT population).

	FF/VI	UC
	*N* = 1396	*N* = 1403
Male, *n* (%)	698 (50)	732 (52)
Age, years		
Mean (SD)	66.6 (9.9)	66.7 (9.9)
Range	40–93	40–91
BMI, kg/m^2^	*n* = 1109	*n* = 1122
Mean (SD)	27.9 (6.53)	27.7 (6.39)
CAT score	*n* = 1394	*n* = 1402
Mean (SD)	21.6 (8.9)	21.9 (8.8)
⩾10, *n* (%)	1243 (89)	1267 (90)
COPD severity category,^a^ *n* (%)	*n* = 1098	*n* = 1101
No airflow obstruction	132 (12)	136 (12)
GOLD grade 1 or 2	652 (59)	641 (58)
GOLD grade 3 or 4	314 (29)	324 (29)
Current medical conditions, *n* (%)		
Any condition	1069 (77)	1076 (77)
Cardiovascular risk factors	720 (52)	728 (52)
Vascular disorders	688 (49)	675 (48)
Cardiac disorders	353 (25)	367 (26)
Respiratory disorders (asthma)	316 (23)	293 (21)
COPD exacerbations in the 12 months prior to randomisation		
Number of moderate/severe exacerbations, mean (SD)	1.98 (1.90)	2.04 (2.08)
⩾1 exacerbation, *n* (%)	1135 (81)	1134 (81)
COPD exacerbations^b^ requiring oral/systemic corticosteroids and/or antibiotics (not requiring hospitalisation), *n* (%)		
0	277 (20)	291 (21)
1	459 (33)	455 (32)
2	288 (21)	269 (19)
>2	372 (27)	388 (28)
COPD exacerbations^b^ requiring hospitalisation, *n* (%)		
1	73 (5)	82 (6)
2	10 (<1)	5 (<1)
>2	11 (<1)	5 (<1)

a Based on available data for forced expiratory volume in 1 s and forced vital capacity.

b In the 12 months prior to randomisation.

BMI, body mass index; CAT, COPD Assessment Test; COPD, chronic obstructive pulmonary disease; FF/VI, fluticasone furoate/vilanterol; GOLD, Global Initiative for Chronic Obstructive Lung Disease; ITT, intent-to-treat; SD, standard deviation; UC, usual care.

### On-treatment SCCs and associated costs

In the ITT population, 53% of patients in the FF/VI group and 54% in the UC group had more than four all-cause SCCs during the treatment period (Table 2). Mean [standard deviation (SD)] number of all-cause SCCs was 9.7 (13.05) with initiating FF/VI and 9.4 (12.43) with continuing UC. In the PEA population, 55% and 56% of patients had more than four on-treatment all-cause SCCs in the FF/VI and UC groups, respectively. Mean (SD) number of all-cause SCCs was 10.3 (13.77) for FF/VI and 9.6 (12.68) for UC. There were no significant differences in least-squares (LS) mean annual rates of all-cause SCCs for FF/VI *versus* UC in either the ITT population [9.81 *versus* 9.36; rate ratio 1.05; 95% confidence interval (CI) 0.95–1.15; *p* = 0.336] (Table 2 and Supplemental Figure 1) or in the PEA population (10.35 *versus* 9.59; rate ratio 1.08; 95% CI 0.97–1.20; *p* = 0.156).

**Table 2.** Annual rates of on-treatment all-cause and COPD-related SCCs (ITT population).

	All-cause SCCs		COPD-related SCCs	
	FF/VI	UC	FF/VI	UC
	*N* = 1396	*N* = 1403	*N* = 1396	*N* = 1403
All SCCs				
Mean (SD)	9.7 (13.05)	9.4 (12.43)	1.8 (4.70)	1.7 (4.99)
Median (range)	5.0 (0–145)	5.0 (0–116)	0.0 (0–75)	0.0 (0–67)
Patients with SCCs, *n* (%)				
0	257 (18)	262 (19)	911 (65)	907 (65)
1	107 (8)	114 (8)	132 (9)	146 (10)
2	108 (8)	108 (8)	96 (7)	111 (8)
3	85 (6)	84 (6)	65 (5)	73 (5)
4	98 (7)	72 (5)	54 (4)	34 (2)
>4	741 (53)	763 (54)	138 (10)	132 (9)
Outpatient visits				
Mean (SD)	8.2 (11.91)	8.0 (11.26)	1.4 (4.16)	1.3 (4.37)
Range	0–143	0–113	0–67	0–65
A&E visits				
Mean (SD)	0.6 (1.21)	0.7 (1.21)	0.2 (0.55)	0.2 (0.60)
Range	0–11	0–9	0–10	0–7
With admission				
Mean (SD)	0.3 (0.77)	0.3 (0.83)	0.1 (0.45)	0.1 (0.47)
Range	0–9	0–9	0–8	0–6
Without admission				
Mean (SD)	0.3 (0.79)	0.3 (0.75)	0.0 (0.24)	0.0 (0.27)
Range	0–8	0–9	0–4	0–5
With ambulance				
Mean (SD)	0.3 (0.80)	0.3 (0.80)	0.1 (0.40)	0.1 (0.46)
Range	0–11	0–8	0–8	0–7
Without ambulance				
Mean (SD)	0.4 (0.79)	0.4 (0.79)	0.1 (0.31)	0.1 (0.28)
Range	0–8	0–8	0–4	0–4
Hospital admissions				
Mean (SD)	0.9 (2.12)	0.8 (1.39)	0.2 (0.77)	0.2 (0.67)
Range	0–54	0–13	0–16	0–7
LS mean annual rate of SCCs^a^	9.81	9.36	1.57	1.48
Ratio FF/VI *versus* UC (95% CI)	1.05 (0.95–1.15)		1.06 (0.89–1.27)	
* p*-value	0.336		0.488	

a Annual rates of on-treatment all-cause and COPD-related SCCs were analysed using a general linear model, assuming an underlying negative binomial distribution.

A&E, accident and emergency; CI, confidence interval; COPD, chronic obstructive pulmonary disease; FF/VI, fluticasone furoate/vilanterol; ITT, intent-to-treat; LS, least-squares; SCC, secondary care contact; SD, standard deviation; UC, usual care.

In the ITT population, the majority of patients had no on-treatment COPD-related SCCs (65% in each group, ITT population; Table 2). Mean (SD) number of COPD-related SCCs was 1.8 (4.70) with initiating FF/VI and 1.7 (4.99) with continuing UC. In the PEA population, corresponding values for mean (SD) COPD-related SCCs were 2.0 (5.03) for FF/VI and 1.9 (5.36) for UC. LS mean annual rates of COPD-related SCCs were not statistically significantly different for the FF/VI and UC groups in either the ITT (1.57 *versus* 1.48; rate ratio 1.06; 95% CI 0.89–1.27; *p* = 0.488) or PEA populations (1.81 *versus* 1.73; rate ratio 1.05; 95% CI 0.87–1.26; *p* = 0.632).

On-treatment all-cause and COPD-related SCCs were mostly outpatient attendances (Table 2). In the ITT population, mean number of A&E visits for all-cause SCCs was 0.6 in the FF/VI group and 0.7 in the UC group; 0.3 with and 0.4 without an ambulance, in both groups. The corresponding mean number of COPD-related SCC visits to A&E was 0.2 in both groups. Around 60% of all patients did not require admission to hospital during the study (Supplemental Table 2). All-cause hospitalisation LS mean annual rates were similar in both groups (LS mean annual rate FF/VI *versus* UC: 0.87 *versus* 0.82; rate ratio 1.07; 95% CI 0.94–1.22; *p* = 0.307; ITT population). Mean number of days per hospital stay per patient for all-cause admissions was 4.5 in the FF/VI group and 4.2 in the UC group.

*Post hoc* analysis found the mean total cost per patient for all-cause SCC was £2363 for FF/VI and £2302 for UC. Mean total cost per patient for COPD-related SCCs was £484 for FF/VI and £475 for UC, mostly associated with outpatient visits (mean cost FF/VI *versus* UC: £257 *versus* £243) and hospitalisations (mean cost FF/VI *versus* UC: £215 *versus* £221) (Table 3).

**Table 3.** Annual costs of on-treatment all-cause and COPD-related SCCs (ITT population).

Cost, GBP	All-cause SCCs^a^		COPD-related SCCs	
	FF/VI	UC	FF/VI	UC
	*N* = 1396	*N* = 1403	*N* = 1396	*N* = 1403
Total cost	3,299,263	3,229,361	675,762	665,967
Cost per patient				
Total cost				
Mean (SD)	2363 (3420)	2302 (3421)	484 (1371)	475 (1430)
Range	0–28,356	0–33,543	0–16,143	0–20,790
Total outpatient visits				
Mean (SD)	1669 (2369)	1615 (2241)	257 (774)	243 (814)
Range	0–27,900	0–21,576	0–12,462	0–12,090
New outpatient visits				
Mean (SD)	416 (525)	406 (509)	44 (123)	42 (115)
Range	0–4650	0–3720	0–930	0–744
Hospitalisations				
Mean (SD)	615 (1799)	610 (1882)	215 (950)	221 (891)
Range	0–25,566	0–28,152	0–14,748	0–15,954
A&E cost without admission				
Mean (SD)	79 (193)	77 (179)	12 (63)	11 (75)
Range	0–2191	0–2025	0–955	0–1268

a *Post hoc* analysis.

A&E, accident and emergency; COPD, chronic obstructive pulmonary disease; FF/VI, fluticasone furoate/vilanterol; ITT, intent-to-treat; SCC, secondary care contact; SD, standard deviation; UC, usual care.

### On-treatment PCCs and associated costs

In the ITT population, 86% of patients in both groups had more than four all-cause PCCs during the treatment period (Table 4). Mean (SD) number of all-cause PCCs was 21.1 (14.11) with initiating FF/VI and 19.0 (12.93) with continuing UC; similar data were observed for the PEA population. The LS mean annual rates of all-cause PCCs were significantly higher with initiating FF/VI *versus* continuing UC in both the ITT (21.20 *versus* 18.88; rate ratio 1.12; 95% CI 1.05–1.20; *p* < 0.001; Table 4) and PEA populations (21.46 *versus* 19.33; rate ratio 1.11; 95% CI 1.03–1.19; *p* = 0.004). A *post hoc* analysis of all-cause PCCs in the ITT population, excluding study-related visits and miscoded administrative procedures, showed similar findings (LS mean annual rate 15.25 for FF/VI *versus* 13.90 for UC; rate ratio 1.10; 95% CI 1.03–1.17) (Table 4 and Supplemental Figure 1).

**Table 4.** Annual rates of on-treatment all-cause and COPD-related PCCs (ITT population).

	All-cause PCCs		COPD-related PCCs	
	FF/VI	UC	FF/VI	UC
	*N* = 1396	*N* = 1403	*N* = 1396	*N* = 1403
All PCCs				
Mean (SD)	21.1 (14.11)	19.0 (12.93)	2.5 (2.28)	2.5 (2.38)
Median (range)	20.0 (0–95)	18.0 (0–82)	2.0 (0–17)	2.0 (0–15)
Patients with PCCs, *n* (%)				
0	150 (11)	150 (11)	276 (20)	291 (21)
1	8 (<1)	8 (<1)	272 (19)	265 (19)
2	11 (<1)	17 (1)	287 (21)	273 (19)
3	11 (<1)	14 (<1)	215 (15)	203 (14)
4	15 (1)	11 (<1)	142 (10)	134 (10)
>4	1201 (86)	1203 (86)	204 (15)	237 (17)
General practitioner				
Mean (SD)	8.9 (8.09)	8.3 (7.44)	1.4 (1.78)	1.4 (1.82)
Range	0–94	0–54	0–16	0–14
Nurse				
Mean (SD)	5.9 (6.53)	5.5 (5.76)	0.9 (1.17)	0.9 (1.27)
Range	0–43	0–40	0–8	0–11
Other				
Mean (SD)	9.3 (9.08)	7.9 (7.78)	0.8 (1.10)	0.8 (1.16)
Range	0–48	0–53	0–8	0–10
Out-of-hours contacts				
Mean (SD)	0.1 (0.37)	0.1 (0.28)	N/A	N/A
Range	0–4	0–4	N/A	N/A
LS annual mean rate of PCCs^a^	21.20	18.88	2.42	2.46
Ratio FF/VI *versus* UC (95% CI)	1.12 (1.05–1.20)^a^		0.98 (0.92–1.05)	
* p*-value	<0.001		0.622	
LS annual mean rate of PCCs, adjusted^a,b^	15.25	13.90	N/A	N/A
Ratio FF/VI *versus* UC (95% CI)	1.10 (1.03–1.17)^a^		N/A	N/A

a The analysis method was a general linear model assuming an underlying negative binomial distribution with a log-link function and logarithm of time on treatment as an offset variable and adjusted for randomised treatment, baseline COPD maintenance therapy per randomisation stratification, number of moderate/severe COPD exacerbations in the 12 months prior to randomisation, and smoking status at baseline.

b Analysed using a revised categorisation of healthcare professional seen and exclusion of study-related Read codes (*post hoc* analysis); no *p*-value calculated.

CI, confidence interval; COPD, chronic obstructive pulmonary disease; FF/VI, fluticasone furoate/vilanterol; ITT, intent-to-treat; LS, least-squares; N/A, not available; PCC, primary care contact; SD, standard deviation; UC, usual care.

In the ITT population, mean (SD) number of COPD-related PCCs was 2.5 (2.28) with initiating FF/VI and 2.5 (2.38) with continuing UC. In the PEA population, corresponding mean (SD) values for COPD-related PCCs were 2.6 (2.39) for FF/VI and 2.7 (2.47) for UC. LS mean annual rates of COPD-related PCCs were similar between the FF/VI and UC groups in both the ITT (2.42 *versus* 2.46; rate ratio 0.98; 95% CI 0.92–1.05; *p* = 0.622; Table 4 and Supplemental Figure 1) and PEA populations (2.57 *versus* 2.63; rate ratio 0.98; 95% CI 0.91–1.05; *p* = 0.530).

**Figure 1.**
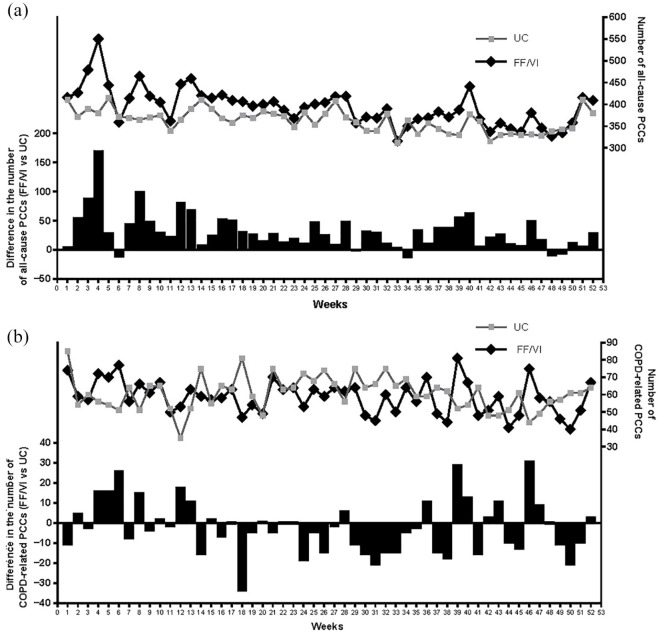
Frequency of on-treatment all-cause PCCs (a) and COPD-related PCCs (b) by study week (ITT population)^a^. ^a^Using a revised categorisation of healthcare professional seen and exclusion of study-related Read codes (*post hoc* analysis). COPD, chronic obstructive pulmonary disease; FF/VI, fluticasone furoate/vilanterol; ITT, intent-to-treat; PCC, primary care contact; UC, usual care.

PCCs by study week showed a higher rate of all-cause PCCs in the first 3 months post-randomisation for patients initiating FF/VI, with pronounced peaks every 4 weeks, which reduced over time [Figure 1(a)]. This pattern was not observed in the UC group, and did not apply to COPD-related PCCs [Figure 1(b)].

PCC costs are summarised in Supplemental Table 3. Mean total cost per patient for all-cause PCCs was higher with initiating FF/VI than continuing UC (mean cost FF/VI *versus* UC: £900 *versus* £811); this pattern was also observed across different HCP categories. However, mean total cost per patient for COPD-related PCCs was similar between groups (mean cost FF/VI *versus* UC: £116 *versus* £114). The total cost for out-of-hours PCCs was also higher in the FF/VI group (£9230) *versus* the UC group (£6471).

### Overall costs of study drug and COPD-related medical care

Total costs of all study drug classes were lower in the FF/VI group than in the UC group (mean overall cost FF/VI *versus* UC: £719,155 *versus* £947,519; mean cost per patient FF/VI *versus* UC: £515 *versus* £675; Table 5). A similar trend was observed across individual study drug subclasses, with lower costs in the FF/VI group *versus* the UC group (Table 5).

**Table 5.** Cost of study drugs (ITT population).

Cost, GBP	FF/VI	UC
	*N* = 1396	*N* = 1403
All classes		
Total cost	719,155	947,519
Total cost per patient		
Mean (SD)	515 (214)	675 (256)
Range	1–1056	5–1221
LABA, *n*	4	31
Total cost per patient, mean (SD)	153 (105)	269 (111)
LAMA, *n*	45	156
Total cost per patient, mean (SD)	211 (136)	327 (128)
LABA + LAMA, *n*	6	27
Total cost per patient, mean (SD)	174 (125)	314 (124)
ICS, *n*	11	71
Total cost per patient, mean (SD)	26 (24)	52 (18)
ICS + LAMA, *n*	5	41
Total cost per patient, mean (SD)	241 (130)	361 (141)
ICS/LABA, *n*	98	416
Total cost per patient, mean (SD)	276 (172)	442 (134)
ICS/LABA/LAMA, *n*	186	827
Total cost per patient, mean (SD)	515 (283)	819 (219)
FF/VI, *n*	622	1
Total cost per patient, mean (SD)	219 (93)	7
FF/VI + LAMA, *n*	813	0
Total cost per patient, mean (SD)	549 (218)	0

FF/VI, fluticasone furoate/vilanterol; ICS, inhaled corticosteroid; ITT, intent-to-treat; LABA, long-acting β_2_-agonist; LAMA, long-acting muscarinic antagonist; SD, standard deviation; UC, usual care.

Direct COPD-related total medical costs (costs for COPD-related HRU plus costs for study medication and rescue medication) were lower in the FF/VI group (£1,596,532) than in the UC group (£1,814,499); corresponding costs per patient were significantly lower with initiating FF/VI *versus* continuing UC (LS geometric mean £806 *versus* £963; ratio of LS geometric means 0.84; 95% CI 0.79–0.88; *p* < 0.001).

### Cost of COPD exacerbations

In the *post hoc* analysis, total cost of moderate/severe exacerbations was £1,238,667 (4847 exacerbations; *n* = 1939), equating to a geometric mean cost of £62 (geometric SD 6.06) per exacerbation.

## Discussion

We used data from SLS COPD to evaluate the impact on HRU of initiating FF/VI 100/25 µg *versus* continuing UC in everyday clinical practice. We found that annual rates of all-cause and COPD-related SCCs were not significantly different for patients initiating FF/VI compared with those continuing UC, and hospitalisation rates and total cost of COPD-related SCCs per patient were similar between groups.

Although the rate of COPD-related PCCs was similar with initiating FF/VI *versus* continuing UC, the rate of all-cause PCCs was significantly higher in the FF/VI group, a finding that was sustained after exclusion of miscoded administrative procedures and study-related visits. A higher annual rate of all-cause PCCs with initiating FF/VI *versus* continuing UC was previously reported in the primary analyses of the SLS COPD and asthma studies.^10,18^ A more in-depth analysis of PCCs by study week in SLS COPD showed a higher rate of all-cause PCCs in the first 3 months post-randomisation for patients who initiated FF/VI, which declined over time. Consequently, higher total costs per patient for all-cause PCCs were observed for FF/VI *versus* UC, but total costs per patient for COPD-related PCCs were similar between the groups.

The cause of the higher rate of all-cause PCCs in the first 3 months after initiating FF/VI is unclear. Possible reasons underlying this observation include: an increased number of patient follow-up visits during the first 3 months post-randomisation; additional scrutiny on the then pre-licensed FF/VI by patients and physicians in the open-label trial; and patients attending visits to switch from FF/VI to an alternative treatment option. The observation of the higher rate of all-cause PCCs in the first 3 months after initiating FF/VI is perhaps not surprising in view of good practice guidance from the New Medicines Service to review patients with long-term conditions who start any new medications. Furthermore, previous research suggests that there is a trend for increased reporting of adverse events with new medications after initiation,^19^ which may lead to additional healthcare contacts. The data available in this study did not allow for the analysis of indirect costs in relation to FF/VI *versus* UC. This is an important limitation given the observed differences between PCC rates in the first 3 months of SLS COPD, which could suggest reduced productivity among patients in the FF/VI arm in this period. However, this analysis was conducted from the perspective of the NHS and did not consider indirect costs.

It should be noted that costs and hospital admission rates were higher in our study than observed in previous studies of HRU in patients with COPD.^20^ However, SLS COPD was designed to recruit a patient population that was representative of the broader population of COPD patients seen in routine clinical practice.^10^ SLS COPD recruited a highly symptomatic, exacerbating, comorbid patient population; such patients are often excluded from participating in traditional efficacy DBRCTs.^7^ As these factors may influence HRU, any comparisons between our findings and HRU data from traditional DBRCTs need to consider potential differences in patient characteristics, particularly with respect to disease severity and comorbid conditions.

The substantially higher number of all-cause *versus* COPD-related SCCs and PCCs observed in our study highlights the burden of non-COPD-related disease in the routine care of patients with COPD. This observation is perhaps not surprising considering that patients with COPD often have other chronic comorbidities, which can result in greater HRU;^21–23^ moreover, the majority (77%) of SLS COPD patients had coexisting conditions. Patients with COPD and cardiovascular disease, for example, have been shown to have higher hospitalisation rates and higher all-cause costs compared with patients with COPD without cardiovascular disease.^22^ Furthermore, a previous study found that the burden of cardiovascular disease in patients with COPD was greater than that associated with COPD itself.^23^ These findings stress the importance of taking a broad approach to COPD management.

In conclusion, these analyses of HRU data from SLS COPD demonstrated that annual rates of all-cause and COPD-related SCCs were similar between initiating FF/VI and continuing UC. Although all-cause PCCs and associated costs were higher in the first 3 months in the FF/VI group, total COPD-related medical costs were substantially lower with initiating FF/VI compared with continuing UC. These findings suggest that FF/VI could offer a less costly alternative to current therapies for the treatment of patients with COPD and exacerbation risk in the UK. With the price of COPD medications and the direct costs of medical care varying in different countries, it would be of interest to explore whether the findings of this cost analysis apply elsewhere.

## Supplemental Material

sj-pdf-1-tar-10.1177_17534666211001013 – Supplemental material for The impact of fluticasone furoate/vilanterol on healthcare resource utilisation in the Salford Lung Study in chronic obstructive pulmonary disease Click here for additional data file. Supplemental material, sj-pdf-1-tar-10.1177_17534666211001013 for The impact of fluticasone furoate/vilanterol on healthcare resource utilisation in the Salford Lung Study in chronic obstructive pulmonary disease by Nawar Diar Bakerly, Dominy Browning, Isabelle Boucot, Jodie Crawford, Sheila McCorkindale, Norman Stein and John P. New in Therapeutic Advances in Respiratory Disease

sj-pdf-2-tar-10.1177_17534666211001013 – Supplemental material for The impact of fluticasone furoate/vilanterol on healthcare resource utilisation in the Salford Lung Study in chronic obstructive pulmonary disease Click here for additional data file. Supplemental material, sj-pdf-2-tar-10.1177_17534666211001013 for The impact of fluticasone furoate/vilanterol on healthcare resource utilisation in the Salford Lung Study in chronic obstructive pulmonary disease by Nawar Diar Bakerly, Dominy Browning, Isabelle Boucot, Jodie Crawford, Sheila McCorkindale, Norman Stein and John P. New in Therapeutic Advances in Respiratory Disease

sj-pdf-3-tar-10.1177_17534666211001013 – Supplemental material for The impact of fluticasone furoate/vilanterol on healthcare resource utilisation in the Salford Lung Study in chronic obstructive pulmonary disease Click here for additional data file. Supplemental material, sj-pdf-3-tar-10.1177_17534666211001013 for The impact of fluticasone furoate/vilanterol on healthcare resource utilisation in the Salford Lung Study in chronic obstructive pulmonary disease by Nawar Diar Bakerly, Dominy Browning, Isabelle Boucot, Jodie Crawford, Sheila McCorkindale, Norman Stein and John P. New in Therapeutic Advances in Respiratory Disease

sj-pdf-4-tar-10.1177_17534666211001013 – Supplemental material for The impact of fluticasone furoate/vilanterol on healthcare resource utilisation in the Salford Lung Study in chronic obstructive pulmonary disease Click here for additional data file. Supplemental material, sj-pdf-4-tar-10.1177_17534666211001013 for The impact of fluticasone furoate/vilanterol on healthcare resource utilisation in the Salford Lung Study in chronic obstructive pulmonary disease by Nawar Diar Bakerly, Dominy Browning, Isabelle Boucot, Jodie Crawford, Sheila McCorkindale, Norman Stein and John P. New in Therapeutic Advances in Respiratory Disease
