# Supplementary material for: The impact of fluticasone furoate/vilanterol on healthcare resource utilisation in the Salford Lung Study in chronic obstructive pulmonary disease
Source: Ther Adv Respir Dis. 2021 Mar 29;15:17534666211001013. doi: 10.1177/17534666211001013 (PMC8013671; doi:10.1177/17534666211001013)
Supplement: sj-pdf-2-tar-10.1177_17534666211001013 – Supplemental material for The impact of fluticasone furoate/vilanterol on healthcare resource utilisation in the Salford Lung Study in chronic obstructive pulmonary disease [file sj-pdf-2-tar-10.1177_17534666211001013.pdf]

Reviewer 1 v.1

Comments to the Author

This is very important and interesting study that assessed the impact of FF/VI on healthcare services use and costs in COPD patients.

The manuscript is very well written and provide the important and necessary data.

Minor comments:

- It would be better to add the currency and currency year in the economic evaluation studies to be able to use the data in future and compare the reports from different regions.

- In the introduction the importance of indirect costs is mentioned but there is no mention of indirect costs in the rest of manuscript, which can be a limitation.

- I suggest adding the time horizon in the text and table, although based on the figure it can be assumed that the costs are per year but there is no mention of the time horizon in the manuscript

- Also, it would be better to add if these costs are from the perspective of the healthcare system or payer or societal.

- In “Table one”, are the numbers of COPD exacerbations requiring hospitalization or oral treatment from the year prior to the study or during study?
