# Supplementary material for: The impact of fluticasone furoate/vilanterol on healthcare resource utilisation in the Salford Lung Study in chronic obstructive pulmonary disease
Source: Ther Adv Respir Dis. 2021 Mar 29;15:17534666211001013. doi: 10.1177/17534666211001013 (PMC8013671; doi:10.1177/17534666211001013)
Supplement: sj-pdf-3-tar-10.1177_17534666211001013 – Supplemental material for The impact of fluticasone furoate/vilanterol on healthcare resource utilisation in the Salford Lung Study in chronic obstructive pulmonary disease [file sj-pdf-3-tar-10.1177_17534666211001013.pdf]

Reviewer 2 v.1

Comments to the Author

Thanks for doing this analysis. There is a need for more cost analysis data. thanks also for attaching supplemental materials.

-As a US reviewer, some of the terminology was different for me (e.g. Read codes)

-As the final analysis seems to come down to differences in medication costs, some of that data would be useful to include in the manuscript vs the supplemental table 4.

-The results section was difficult to read and largely repeated what was in the tables. Could this be streamlined in some way to get to the key points?

-Due to the massive amount of data, I had some trouble linking up some of the information in the text to the tables. I could not find the direct COPD-related medical costs (mentioned on p 12) in any of the tables

Also did not see the p value for total all study drug classes and individual study drug subclasses p 9-10 which were said to be significant

If I am understanding your results correctly, you found that the medicine costs were less, but the outcomes and cost of medical care was essentially the same. is that correct?

I hope you find my comments helpful in revising your manuscript. thanks.
