# Supplementary material for: The impact of fluticasone furoate/vilanterol on healthcare resource utilisation in the Salford Lung Study in chronic obstructive pulmonary disease
Source: Ther Adv Respir Dis. 2021 Mar 29;15:17534666211001013. doi: 10.1177/17534666211001013 (PMC8013671; doi:10.1177/17534666211001013)
Supplement: sj-pdf-4-tar-10.1177_17534666211001013 – Supplemental material for The impact of fluticasone furoate/vilanterol on healthcare resource utilisation in the Salford Lung Study in chronic obstructive pulmonary disease [file sj-pdf-4-tar-10.1177_17534666211001013.pdf]

The impact of fluticasone furoate/vilanterol on healthcare resource utilisation in the Salford Lung Study in chronic obstructive pulmonary disease

SUPPLEMENTARY MATERIAL

SLS COPD study design and patients

The SLS COPD study design has been reported previously.[1] Briefly, the study included patients aged ≥40 years with a general practitioner (GP) diagnosis of COPD, who had experienced at least one COPD exacerbation in the last 3 years and were receiving regular maintenance therapy for COPD. Existing usual care (UC) was defined as treatment with an inhaled corticosteroid (ICS), a long-acting beta$_2$-agonist (LABA), or a long-acting muscarinic antagonist (LAMA) alone or in combination (i.e. long-acting bronchodilator therapy alone [LABA, LAMA, or LABA/LAMA combination]; ICS alone or in combination with a long-acting bronchodilator [ICS, ICS/LABA, or ICS/LAMA]; or triple combination therapy [ICS/LABA + LAMA]). There were no restrictions on smoking history or spirometry values. Among the few exclusion criteria were an exacerbation within the previous 2 weeks and long-term oral glucocorticoid use. Patients were randomised 1:1 (stratified by baseline COPD maintenance therapy and presence or absence of a COPD exacerbation in the previous 12 months) to initiate fluticasone furoate/vilanterol (FF/VI) 100/25 µg or continue with their UC. Patients who were on triple maintenance therapy and were randomised to initiate FF/VI were prescribed their usual LAMA therapy in addition to FF/VI. Treatment modifications were permitted in both treatment groups throughout the study. Follow-up was for 12 months.

Cost calculations

The total all-cause cost for secondary care was calculated as: (total cost of all-cause hospitalisations) + (total cost of all-cause outpatient visits) + (total cost of all-cause A&E visits without inpatient admission).

The total cost per exacerbation was calculated as: (total cost of primary care contact, including seen by GP, nurse, and other) + (total cost of hospitalisations) + (total cost of A&E visits, including with inpatient admission, without inpatient admission and ambulance use) + (total cost of outpatient visits, including being seen by respiratory specialist) + (total cost of out-of-hours contacts, including being seen by GP and nurse) + (steroids and antibiotics taken for exacerbation).

Supplemental Table 1. Summary of unit costs for HRU and study medications in SLS COPD.

Costs for healthcare contacts	Unit cost, GBP	Cost source and assumptions
PCC, GP	45.00	PSSRU 2015[a]; per patient face-to-face contact lasting 11.7 min
PCC, Nurse	25.00	PSSRU 2015[a]; per patient consultation (including qualification costs)
PCC, Other	38.00	PSSRU 2015[a]; assumed equal to the costs for a dietitian
Out-of-hours contact, GP	90.00	PSSRU 2015[a]; per patient face-to-face contact lasting 11.7 min. Average GP home visit time is 11.4 min, + 12 min travel time; average time for a GP consultation within a practice is 11.7 min, at a cost of £45; total cost = £45[a] × 23.4/11.7 = £90
Out-of-hours contact, Nurse	59.68	PSSRU 2015[a]; per patient consultation (including qualification costs). Average nurse home visit time is 25 min, + 12 min travel time; average time for a nurse consultation within a practice is 15.5 min, at a cost of £25; total cost = £25[a] × 37/15.5 = £59.68
COPD-related day in hospital	134.83	2014/2015 National Tariff Payment System: Annex 5A, National Prices[b] Code DZ21K—Chronic Obstructive Pulmonary Disease or Bronchitis without NIV without Intubation without CC (nonelective spell tariff for 12 days)
CVD-related day in hospital	164.58	2014/2015 National Tariff Payment System: Annex 5A, National Prices[b] Code EB04I Hypertension without CC (nonelective spell tariff for 12 days)
Outpatient visit seen by respiratory specialist	186.00	2014/2015 National Tariff Payment System: Annex 5A, National Prices[b]—All NHS trusts and NHS foundation trusts—Outpatient Attendances Data—consultant led; treatment function 340
Outpatient visit seen by cardiologist	164.00	2014/2015 National Tariff Payment System: Annex 5A, National Prices[b] All NHS trusts and NHS foundation trusts—Outpatient Attendances Data—Cardiologist; treatment function 320
A&E visit with inpatient admission	246.00	NHS Reference Costs 2014 to 2015[c]—NHS trusts and NHS foundation trusts—Emergency Medicine. Code T01A Emergency Medicine, Any Investigation. Average from categories 1–5
A&E visit without inpatient admission	214.00	NHS Reference Costs 2014 to 2015[c] —NHS trusts and NHS foundation trusts—Emergency Medicine. Code T01NA Emergency Medicine, Any Investigation. Average from categories 1–5
Ambulance use for an A&E visit	99.00	PSSRU 2015[a]
LABA	0.9093	Weighted average for class based on UK sales data[d]
LAMA	1.0653	Weighted average for class based on UK sales data[d]
LABA/LAMA	1.0833	Weighted average for class based on UK sales data[d]
ICS	0.1647	Weighted average for class based on UK sales data[d]
ICS/LABA	1.3613	Weighted average for class based on UK sales data[d]
ICS/LAMA	1.2300	Weighted average for class based on UK sales data[d]
ICS/LABA/LAMA	2.4267	Weighted average for class based on UK sales data[d]
FF/VI	0.7333	Weighted average for class based on UK sales data[d]
FF/VI + LABA	1.6426	Weighted average for class based on UK sales data[d]
FF/VI + LAMA	1.7987	Weighted average for class based on UK sales data[d]
FF/VI + ICS	0.8980	Weighted average for class based on UK sales data[d]
FF/VI + LABA/LAMA	1.8166	Weighted average for class based on UK sales data[d]
FF/VI + ICS/LABA/LAMA[e]	3.1600	Weighted average for class based on UK sales data[d]

[a]PSSRU 2015 data.[2]

[b]2014/2015 National Tariff Payment System: Annex 5A, National Prices [NHS England Publications Gateway].[3]

[c] NHS Reference Costs 2014 to 2015.[4]

[d]MIMS.[5]

[e]Twelve patients across both randomised treatment arms took multiple treatments of the same class at the same time.

A&E, Accident and Emergency; CC, complication and comorbidity; COPD, chronic obstructive pulmonary disease; CVD, cardiovascular disease; FF/VI, fluticasone furoate/vilanterol; GP, general practitioner; HRU, healthcare resource utilisation; ICS, inhaled corticosteroid; LABA, long-acting beta$_2$-agonist; LAMA, long-acting muscarinic antagonist; MIMS, Monthly Index of Medical Specialities; NHS, National Health Service; NIV, noninvasive ventilation; PCC, primary care contact; PSSRU, Personal Social Services Research Unit; SLS, Salford Lung Study.

Supplemental Table 2. On-treatment all-cause and COPD-related hospital admissions (ITT population).

	**All-cause hospital admissions**		**COPD-related hospital admissions[b]**	
	**FF/VI** ***N*=1396**	**UC** ***N*=1403**	**FF/VI** ***N*=1396**	**UC** ***N*=1403**
Patient admissions, *n* (%)				
0	821 (59)	840 (60)	1167 (84)	1178 (84)
1	315 (23)	291 (21)	173 (12)	155 (11)
2	122 (9)	134 (10)	33 (2)	44 (3)
3	69 (5)	66 (5)	14 (1)	15 (1)
4	31 (2)	30 (2)	7 (<1)	8 (<1)
>4	38 (3)	42 (3)	2 (<1)	3 (<1)
Patients hospitalised ≥1 times, *n*	575	563	229	225
Total number of hospital stays	1206	1138	335	338
LS mean annual rate[a]	0.87	0.82	0.23	0.23
Ratio (95% CI)	1.07 (0.94–1.22)		1.01 (0.82–1.23)	
p-value	0.307		0.961	
Average number of days per stay	4.5	4.4	5.7	5.8
Total number of days in hospital per patient	*n*=575	*n*=560	*n*=229	*n*=225
Mean (SD)	9.4 (17.41)	8.8 (16.26)	8.4 (14.2)	8.7 (13.0)
Median (range)	4.0 (1–175)	3.0 (1–126)	3.0 (1–105)	4.0 (1–107)
Average number of days per stay per patient	*n*=575	*n*=560	*n*=229	*n*=225
Mean (SD)	4.5 (7.03)	4.2 (8.63)	5.7 (9.3)	5.3 (6.0)
Median (range)	2.0 (1–76)	2.0 (1–120)	3.0 (1–92)	3.0 (1–35)

[a]The analysis method was general linear model assuming an underlying negative binomial distribution with a log-link function and logarithm of time on treatment as an offset variable and adjusted for randomised treatment, baseline COPD maintenance therapy per randomisation stratification, number of moderate/severe COPD exacerbations in the 12 months prior to randomisation, and smoking status at baseline.

[b]Post-hoc analysis.

CI, confidence interval; COPD, chronic obstructive pulmonary disease; FF/VI, fluticasone furoate/vilanterol; ITT, intent-to-treat; LS, least-squares; SD, standard deviation; UC, usual care.

Supplemental Table 3. Costs of on-treatment all-cause and COPD-related PCCs (ITT population).

	All-cause PCCs		COPD-related PCCs	
**Cost, GBP**	**FF/VI** **_N_=1396**	**UC** **_N_=1403**	**FF/VI** **_N_=1396**	**UC** **_N_=1403**
Total cost	1,256,366	1,137,422	161,556	159,535
Cost per patient, mean (SD)	900 (651)	811 (608)	116 (116)	114 (118)
Cost by HCP type, mean (SD)				
General practitioner	398 (364)	373 (335)	63 (80)	61 (82)
Nurse	147 (163)	137 (144)	22 (29)	24 (32)
Other	355 (345)	301 (296)	30 (42)	29 (44)
Total cost for out-of-hours contacts	9230	6471	N/A	N/A
Cost per patient, mean (SD)	7 (29)	5 (22)	N/A	N/A

COPD, chronic obstructive pulmonary disease; FF/VI, fluticasone furoate/vilanterol; HCP, healthcare professional; ITT, intent-to-treat; N/A, not available; PCC, primary care contact; SD, standard deviation; UC, usual care.

Supplemental Figure 1. Mean annual rates of on-treatment all-cause and COPD-related SCCs and PCCs (ITT population).

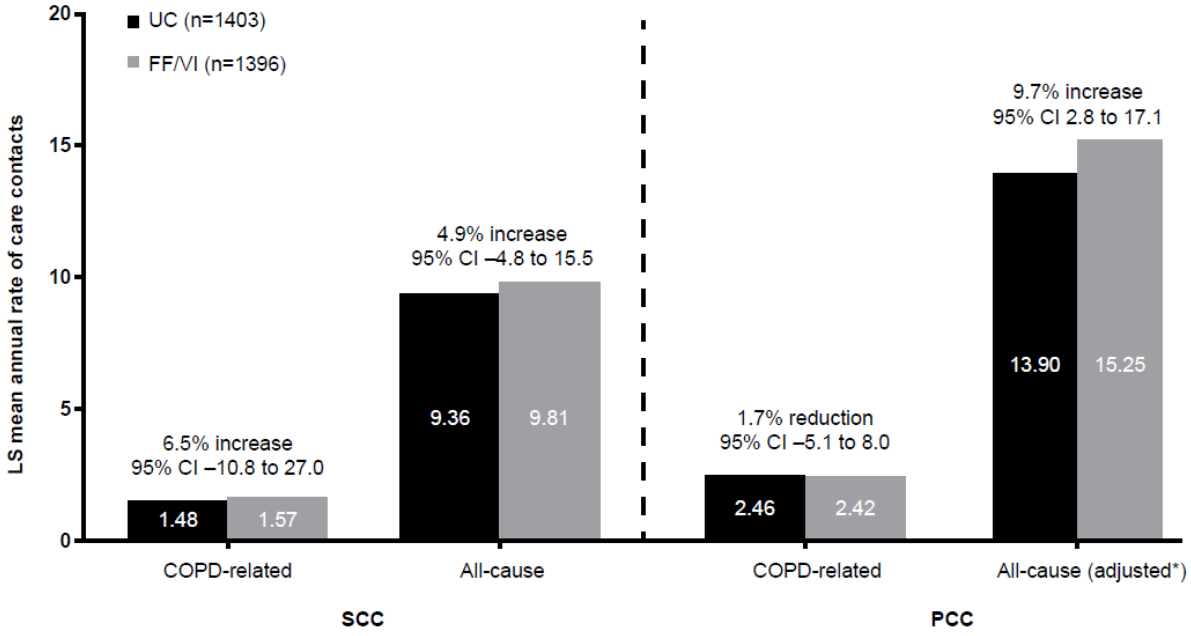

*Using a revised categorisation of HCP seen and exclusion of study-related Read codes (*post-hoc* analysis).

CI, confidence interval; COPD, chronic obstructive pulmonary disease; FF/VI, fluticasone furoate/vilanterol; HCP, healthcare professional; ITT, intent-to-treat; LS, least-squares; PCC, primary care contact; SCC, secondary care contact; UC, usual care.

References

1. Vestbo J, Leather D, Diar Bakerly N, et al. Effectiveness of fluticasone furoate-vilanterol for COPD in clinical practice. *N Engl J Med* 2016; 375: 1253–1260.
2. Curtis L, Burns A. Unit costs of health and social care 2015. Personal Social Services Research Unit, University of Kent, Canterbury. 2015. https://www.pssru.ac.uk/project-pages/unit-costs/unit-costs-2015/ (accessed 13 May 2019).
3. NHS England Publications Gateway Reference 00883. 2014/2015 National tariff payment system. Annex 5A: national prices. https://assets.publishing.service.gov.uk/government/uploads/system/uploads/attachment_data/file/300551/Annex_5A_National_Prices.xlsx (accessed 13 May 2019).
4. NHS Reference Costs 2014 to 2015. https://www.gov.uk/government/publications/nhs-reference-costs-2014-to-2015 (accessed 13 May 2019).
5. Monthly Index of Medical Specialities. November 2015. https://www.mims.co.uk/ (accessed 13 May 2019).
