# Supplementary material for: The impact of fluticasone furoate/vilanterol on healthcare resource utilisation in the Salford Lung Study in chronic obstructive pulmonary disease
Source: Ther Adv Respir Dis. 2021 Mar 29;15:17534666211001013. doi: 10.1177/17534666211001013 (PMC8013671; doi:10.1177/17534666211001013)
Supplement: sj-pdf-1-tar-10.1177_17534666211001013 – Supplemental material for The impact of fluticasone furoate/vilanterol on healthcare resource utilisation in the Salford Lung Study in chronic obstructive pulmonary disease [file sj-pdf-1-tar-10.1177_17534666211001013.pdf]

Dr Philip Shaw
Managing Editor, *Therapeutic Advances in Respiratory Disease*
respiratory@sagepub.co.uk

22 March 2021

Re Manuscript ID: TAR-20-236
Title: The impact of fluticasone furoate/vilanterol on healthcare resource utilisation in the Salford Lung Study in chronic obstructive pulmonary disease

Dear Dr Shaw,
On behalf of my co-authors, I would like to thank you for considering our manuscript for publication and providing us with the opportunity to address comments from the peer reviewers. Please find enclosed our point-by-point reply to the comments (additions to manuscript text are underlined and deletions are in strikethrough text) and our revised manuscript with changes tracked. I hope you will now consider our article suitable for publication in *Therapeutic Advances in Respiratory Disease*. Please do let me know if anything further is required. I look forward to receiving your decision.

Yours sincerely,
Nawar Diar Bakerly

Nawar Diar Bakerly, MD
Department of Respiratory Medicine
Salford Royal NHS Foundation Trust
Stott Lane, Salford
M6 8HD, UK
Tel: +44 (0) 161 206 3187
E-mail: nawar.bakerly@srft.nhs.uk

Reviewers' comments:

Reviewer #1:

This is very important and interesting study that assessed the impact of FF/VI on healthcare services use and costs in COPD patients.

The manuscript is very well written and provide the important and necessary data.

Author response: We thank the reviewer for their kind remarks and considered comments on the manuscript.

It would be better to add the currency and currency year in the economic evaluation studies to be able to use the data in future and compare the reports from different regions.

Author response: We agree with the reviewer that the manuscript would benefit from the inclusion of currency year and have added the following to the methods section:

Page 7: *"All costs (except unit costs) were to the nearest whole pound (GBP [£], 2014–15)"*

In the introduction the importance of indirect costs is mentioned but there is no mention of indirect costs in the rest of manuscript, which can be a limitation.

Author response: The reviewer has raised an important point and we have added the following to the discussion on the issue of indirect costs:

Page 13: *"The data available in this study did not allow for the analysis of indirect costs in relation to FF/VI versus UC. This is an important limitation given the observed differences between PCC rates in the first 3 months of SLS COPD, which could suggest reduced productivity among patients in the FF/VI arm in this period. However, this analysis was conducted from the perspective of the NHS and did not consider indirect costs."*

I suggest adding the time horizon in the text and table, although based on the figure it can be assumed that the costs are per year but there is no mention of the time horizon in the manuscript

Author response: We thank the reviewer for highlighting this omission, we have added the following to the manuscript text:

Page 7: *"Results are reported for a time horizon of one year and from an NHS perspective."*

We have additionally included *"Annual"* in the following table titles:

Table 2 (Page 22): *"**Table 2**. Annual ~~R~~rates of on-treatment all-cause and COPD-related SCCs (ITT population)."*

Table 3 (Page 24): *"**Table 3**. Annual ~~C~~costs of on-treatment all-cause and COPD-related SCCs (ITT population)."*

Table 4 (Page 25): *"**Table 4**. Annual ~~R~~rates of on-treatment all-cause and COPD-related PCCs (ITT population)."*

Also, it would be better to add if these costs are from the perspective of the healthcare system or payer or societal.

Author response: We thank the reviewer for highlighting this omission, we have added the following to the manuscript text:

Page 7: *“Results are reported for a time horizon of one year and from an NHS perspective.”*

In “Table one”, are the numbers of COPD exacerbations requiring hospitalization or oral treatment from the year prior to the study or during study?

Author response: We added a footnote to the table to clarify this point:

Table 1 (Page 20): *“[b]In the 12 months prior to randomisation.”*

Reviewer #2:

Thanks for doing this analysis. There is a need for more cost analysis data. thanks also for attaching supplemental materials.

Author response: We thank the reviewer for taking the time to review our manuscript and for providing thoughtful comments on our work.

As a US reviewer, some of the terminology was different for me (e.g. Read codes)

Author response: The reviewer makes a valid point that the language used in the manuscript is targeted more towards a UK audience. However, as the SLS was conducted in UK primary care, we feel it is important to use language consistent with this setting and, as we note in the discussion, costs vary between countries and further analyses will be necessary to test the generalisability of our results outside of the UK. Given that our study is certainly most applicable to the UK we have not amended the language in this instance.

As the final analysis seems to come down to differences in medication costs, some of that data would be useful to include in the manuscript vs the supplemental table 4.

Author response: We thank the reviewer for their suggestion and have moved Supplemental Table A4 from the supplement to the main text as Table 5 (page 27; placeholder on page 12)

The results section was difficult to read and largely repeated what was in the tables. Could this be streamlined in some way to get to the key points?

Author response: We are grateful to the reviewer for this comment. In an effort to streamline the results section, we have removed reference to “*median (range)”* values from the text (as these are available in the tables) but maintained the reference to mean values:

Page 8: *“Mean (standard deviation [SD]) number of all-cause SCCs was 9.7 (13.05) with initiating FF/VI and 9.4 (12.43) with continuing UC~~; median (range) values were 5.0 (0–145) and 5.0 (0–116), respectively~~. In the PEA population, 55% and 56% of patients had more than four on-treatment all-cause SCCs in the FF/VI and UC groups, respectively. Mean (SD) number of all-cause SCCs was 10.3 (13.77) for FF/VI and 9.6 (12.68) for UC~~; median (range) values were 5.0 (0–145) and 5.0 (0–116), respectively~~.”*

Page 9: *“Mean (SD) number of COPD-related SCCs was 1.8 (4.70) with initiating FF/VI and 1.7 (4.99) with continuing UC~~; median (range) values were 0.0 (0–75) and 0.0 (0–67), respectively~~. In the PEA population, corresponding values for mean (SD) COPD-related SCCs were 2.0 (5.03) for FF/VI and 1.9 (5.36) for UC~~; median (range) values were 0.0 (0–75) and 0.0 (0–67), respectively~~.*

Page 10: *"Mean (SD) number of all-cause PCCs was 21.1 (14.11) with initiating FF/VI and 19.0 (12.93) with continuing UC~~; median (range) values were 20.0 (0–95) and 18.0 (0–82), respectively~~; similar data were observed for the PEA population."*

"In the ITT population, mean (SD) number of COPD-related PCCs was 2.5 (2.28) with initiating FF/VI and 2.5 (2.38) with continuing UC~~; median (range) values were 2.0 (0–17) and 2.0 (0–15), respectively~~."

Pages 10–11: *"In the PEA population, corresponding mean (SD) values for COPD-related PCCs were 2.6 (2.39) for FF/VI and 2.7 (2.47) for UC~~; median (range) values were 2.0 (0–17) and 2.0 (0–15), respectively~~."*

Additionally, we removed the mention of A&E visits with and without an ambulance as this is a secondary point, no difference was observed between the groups and the data are included in the corresponding table:

Page 9: *"The corresponding mean number of COPD-related SCC visits to A&E was 0.2 in both groups~~; mean number of these visits with and without an ambulance was 0.1 for both categories in both groups~~."*

Due to the massive amount of data, I had some trouble linking up some of the information in the text to the tables. I could not find the direct COPD-related medical costs (mentioned on p12) in any of the tables

Author response: We hope that with our revisions we have been able to streamline the manuscript and make it easier to follow. Regarding direct COPD-related medical costs, as these are two values fully reported in the text, we did not add them to any of the tables. We have included a further reference to Table 5 in the text for clarity:

Pages 11–12: *"A similar trend was observed across individual study drug subclasses, with lower costs in the FF/VI group versus the UC group (Table 5)."*

Also did not see the p value for total all study drug classes and individual study drug subclasses p 9-10 which were said to be significant

Author response: We apologise to the reviewer for any confusion, however, we did not describe the differences in total all study drug classes and individual study drug subclasses as "significant", we only noted that costs were lower for the FF/VI group than the UC group as follows:

Pages 11–12: *"Total costs of all study drug classes were lower in the FF/VI group than in the UC group (mean overall cost FF/VI vs. UC: £719,155 vs. £947,519; mean cost per patient FF/VI vs. UC: £515 vs. £675; ~~Supplemental Material~~ Table 5~~A4~~). A similar trend was observed across individual study drug subclasses, with lower costs in the FF/VI group versus the UC group."*

As such, we have not included any p-value in the manuscript text here.

If I am understanding your results correctly, you found that the medicine costs were less, but the outcomes and cost of medical care was essentially the same. is that correct?

Author response: The reviewer is correct, PCC costs were higher in the first three months of the SLS for the FF/VI group vs the UC group, however SCC costs were similar and medication costs were lower for FF/VI, resulting in a substantially lower overall cost. This was summarised in the discussion as follows:

Pages 14–15: *“In conclusion, these analyses of HRU data from SLS COPD demonstrated that annual rates of all-cause and COPD-related SCCs were similar between initiating FF/VI and continuing UC. Although all-cause PCCs and associated costs were higher in the first 3 months in the FF/VI group, total COPD-related medical costs were substantially lower with initiating FF/VI compared with continuing UC.”*

Additional corrections of typographical errors

Page 6: *“Prices were derived ~~from~~ for general practice resource use…”*

Table A1, row 5 (Out-of-hours contact, GP), column 3 (Cost source and assumptions): *“…average time for a GP consultation within a practice is 11.7 min, at a cost of £~~90~~45; total cost = £45[a] × 23.4/11.7 = £90”*

Table A1, row 9 (Outpatient visit seen by respiratory specialist), column 3 (Cost source and assumptions): *“…consultant led; treatment function 3~~2~~40”*

Table A1, row 10 (Outpatient visit seen by cardiologist), column 3 (Cost source and assumptions): *“…Cardiologist; treatment function 3~~4~~20”*

Table A1, row 11 (A&E visit with inpatient admission), column 3 (Cost source and assumptions): *“NHS Reference Costs 2014 to 2015[c] ~~2014/2015 National Tariff Payment System: Annex 5A, National Prices[b]~~…”*

Table A1, row 12 (A&E visit without inpatient admission), column 3 (Cost source and assumptions): *“NHS Reference Costs 2014 to 2015[c] ~~2014/2015 National Tariff Payment System: Annex 5A, National Prices[b]~~…”*

Table A1, rows 14–26, column 3 (Cost source and assumptions): *“Weighted average for class based on UK sales data[~~c~~d]”*

Table A1, row 26, column 1: *“FF/VI + ICS/LABA/LAMA[~~d~~e]”*

Added to Table A1 footnote: *“[c] NHS Reference Costs 2014 to 2015.[4]”*

Added to Supplement references: “*4. NHS Reference Costs 2014 to 2015. https://www.gov.uk/government/publications/nhs-reference-costs-2014-to-2015 (accessed 13 May 2019).*”
